# The imperative for systems thinking to promote access to medicines, efficient delivery, and cost-effectiveness when implementing health financing reforms: a qualitative study

**DOI:** 10.1186/s12939-017-0550-x

**Published:** 2017-03-21

**Authors:** Tom Achoki, Abaleng Lesego

**Affiliations:** 1 Department of Global Health, University of Washington, Seattle, Washington USA; 2 Centre for Pharmaceutical Policy and Regulation, Utrecht University, Utrecht, Netherlands; 3 Botswana University of Maryland Baltimore School of Medicine Health Initiative, Gaborone, Botswana

**Keywords:** Health financing, Efficiency, Access to medicines, Health insurance, Health systems

## Abstract

**Background:**

Health systems across Africa are faced with a multitude of competing priorities amidst pressing resource constraints. Expansion of health insurance coverage offers promise in the quest for sustainable healthcare financing for many of the health systems in the region. However, the broader policy implications of expanding health insurance coverage have not been fully investigated and contextualized to many African health systems.

**Methods:**

We interviewed 37 key informants drawn from public, private and civil society organizations involved in health service delivery in Botswana. The objective was to determine the potential health system impacts that would result from expanding the health insurance scheme covering public sector employees. Study participants were selected through purposeful sampling, stakeholder mapping, and snowballing. We thematically synthesized their views, focusing on the key health system areas of access to medicines, efficiency and cost-effectiveness, as intermediate milestones towards universal health coverage.

**Results:**

Participants suggested that expansion of health insurance would be characterized by increased financial resources for health and catalyze an upsurge in utilization of health services particularly among those with health insurance cover. As a result, the health system, particularly within the private sector, would be expected to see higher demand for medicines and other health technologies. However, majority of the respondents cautioned that, realizing the full benefits of improved population health, equitable distribution and financial risk protection, would be wholly dependent on having sound policies, regulations and functional accountability systems in place. It was recommended that, health system stewards should embrace efficient and cost-effective delivery, in order to make progress towards universal health coverage.

**Conclusion:**

Despite the prospects of increasing financial resources available for health service delivery, expansion of health insurance also comes with many challenges. Decision-makers keen to achieve universal health coverage, must view health financing reform through the holistic lens of the health system and its interactions with the population, in order to anticipate its potential benefits and risks. Failure to embrace this comprehensive approach, would potentially lead to counterproductive results.

## Background

Recently, many African countries have reported steady progress in tackling the major health challenges that ravaged the region [1–3]. A significant amount of resources from various sources have been invested to implement comprehensive health programs aimed at addressing HIV/AIDS; malaria and tuberculosis, among other priority health conditions in the region [4, 5]. Key maternal and child health interventions have also been rapidly scaled up and these measures have been suggested to have contributed significantly to the recent improvements in child survival [1–4].

With many countries striving to consolidate their recent health gains, the question on how to tackle the increasing health needs in the face of emerging non-communicable diseases is becoming increasingly important [2, 3]. This has compelled a vibrant debate on sustainability, mainly centered on the aspiration of universal health coverage (UHC) and the means to finance such an endeavor in low- and middle- income countries, where resources are scarce. UHC is about all people having access to healthcare services they need without suffering financial hardship [4, 6]. According to the World Health Organization (WHO), the health services should be of high quality and include, prevention, promotion, treatment and rehabilitation [6].

However, majority of African health systems are resource constrained and heavily reliant on donor funds to finance different aspects of health service delivery. This financing situation is largely untenable in the long-term, given the recent plateauing of financial resource inflows to recipient countries and the unpredictable nature of donor funding [2, 3, 5]. In response, there have been concerted efforts in many countries seeking ways to align their health financing strategies with the ambitious policy aspirations of UHC. Key to this goal, is to unlock the potential of domestic resources as a means towards sustainable health financing [4–6]. Domestic resources are perceived to be more predictable, unlike donor funds that often shift with the changing whims of funders, with little regard to national priorities. In addition, it is often opined that domestic resources are likely to strengthen the sense of ownership and therefore accountability in health programming at different levels of implementation [1, 4, 6]. To effectively mobilize domestic resources for health, countries should have functional structures to collect and pool resources in order to strategically purchase appropriate health goods and services [4–6].

Ultimately, the overarching policy objective of any national health financing strategy is to raise sufficient funds in ways that ensure the population in need can access quality health services without undue financial pressure [4–6]. Kutzin [7] further elaborates that the concept of UHC is not only achieved through financial risk protection, but closely linked to effective coverage, such that the beneficiaries who receive the health services should experience health gains as a result. Therefore, to be congruent with the UHC aspirations, health financing reforms should not only be viewed through the narrow lens of providing financial risk protection alone, but also seek to expand effective coverage and improve population health outcomes [4, 6, 7]. Obviously, to achieve these objectives, health financing reforms should also be aligned with the desirable attributes of efficiency, equity, transparency and accountability [4, 7].

These facts clarify that any health financing reform needs to be investigated holistically, paying specific attention to the complex interactions with different components of the broader health system, and how that affects progress towards UHC. Often, health financing reforms have both intended and unintended consequences, which health system stewards need to anticipate and address accordingly. Failure to consider and appreciate this holistic view could lead to disastrous consequences that have far reaching implications on population health [4, 7].

Agyepong and Adjei [8] in a case study describing the policy development and implementation of Ghana’s National Health Insurance Scheme (NHIS), point out to the complex interactions and power plays among different actors in the politics of health systems reform. In this case, it was clear that the available technical evidence was not necessarily used to inform important decisions, but rather the persuasions of the strong and dominant political actors. Therefore, the resultant health insurance scheme that was adopted still faces many challenges. For example, Addae-Korankye [9] explains that, within NHIS there are still economic and financial barriers and membership is skewed against the poor and marginalized groups. Fusheini [10] further identifies governance, operational, administrative and financial challenges as factors leading to service delivery challenges within the Ghana NHIS. Similar challenges have been reported by Chuma and Okungu [11] in assessing Kenya’s efforts to introduce a national health insurance scheme.

Further, Tangcharoensathien et al. [12] recognize that pre-payments to health insurance schemes in some cases does not guarantee financial risk protection. This is particularly true in small health insurance pools where there is inadequate cross subsidization among the membership. However, despite these challenges, a number of countries such as Thailand, Rwanda and the Philippines have made steady progress towards UHC, by expanding health insurance coverage [4, 13]. In addition, Knaul et al. [14] provides a compelling case for expansion of health insurance coverage as a remedy for catastrophic health spending and impoverishment of households in Mexico.

This paper is based on the perspectives of health system stakeholders in Botswana, a middle income country in Africa, which sought to expand the public sector employees’ health insurance scheme as a step towards UHC. In our analysis, we specifically focused on access to medicines, efficiency and cost-effectiveness of health service delivery as intermediate milestones towards UHC. This was motivated by the fact that, medicines and other health technologies have been shown to be a major driver of health expenditure in many low- and middle- income countries, and that significant resources could be saved through cost-effective selection and efficient management of the same [4]. We have further mainstreamed important aspects of the health system performance such as quality, equity, transparency and accountability in our analysis. Of course, we recognize that there are other equally important aspects of the health systems that warrant such investigation, but have elected to focus on these considering their relevance to the context of low- and middle-income countries [4, 6, 7].

## Methods

This was a qualitative study that sought to determine the potential health systems impacts that would result from the proposed expansion of the public sector employees’ health insurance scheme as a pathway towards UHC in Botswana. It was part of a larger study that sought to understand the demand and uptake of health insurance among the public sector employees in the country.

### Setting

In Botswana, the health system follows a decentralized structure with varying levels of autonomy at the district level. The national Ministry of Health (MOH) is the central planning and policy formulation unit, with the overall responsibility of coordinating and supervising district level implementers to achieve the national health policy objectives [15, 16].

**Fig. 1:**
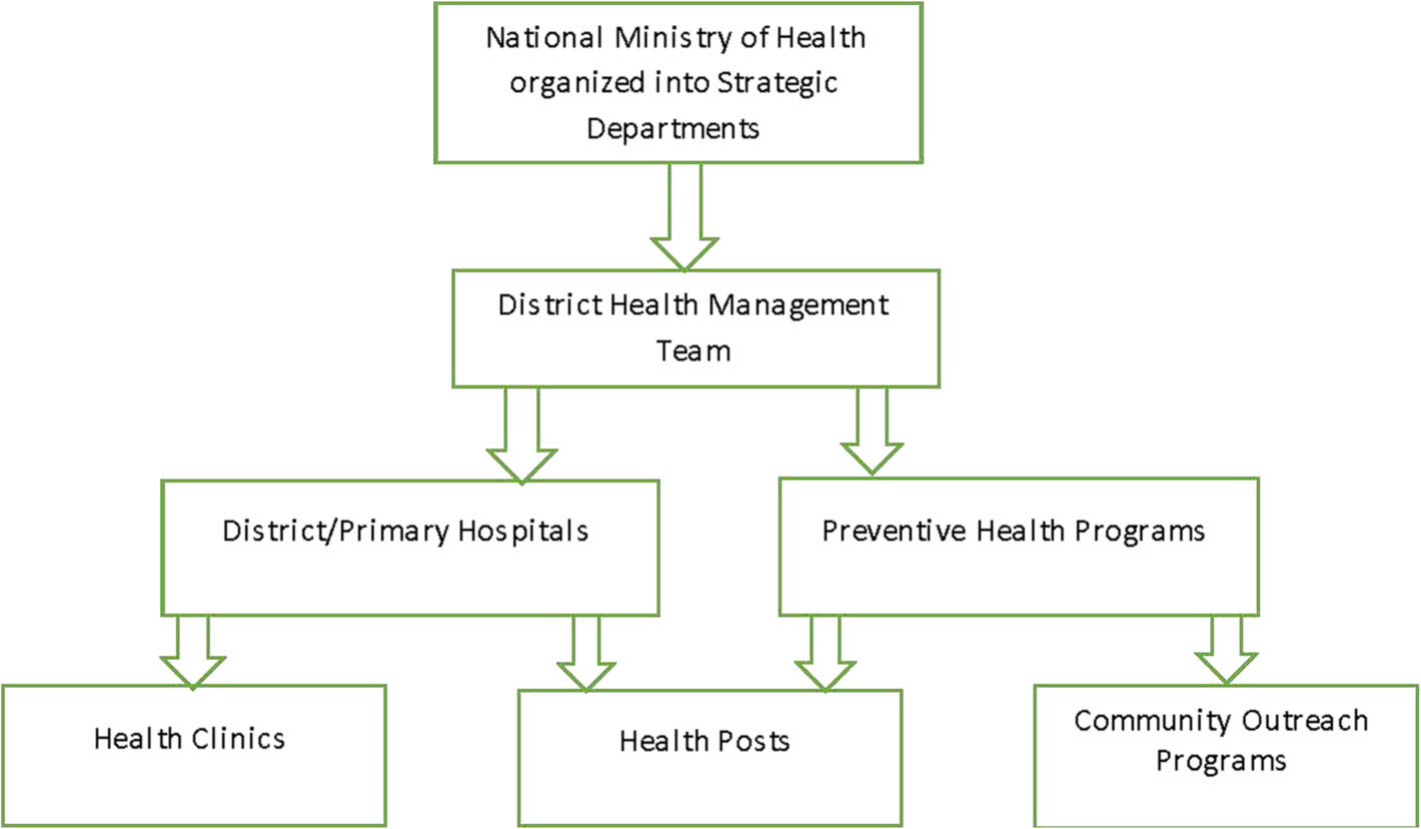
Ministry of Health Organizational Structure. The key Ministry of Health, organizational structures charged with policy setting and implementation

The district health system is comprised of many actors, drawn from the public, private and civil society, all working together to deliver health services. The district is the core implementation unit providing health services based on the principles of the primary health care (PHC) [15, 16]. Within the district, there are different levels of health facilities that form the service delivery chain. These range from clinics and health posts, to primary and district hospitals. The latter form the first referral point within the district health system, offering a range of basic specialist support to the clinics and health posts (that mainly provide preventive health services) [16, 17]. There are also private providers at the district level, some of which have entered into collaborative arrangements with the public sector to provide health services. At the pinnacle of the referral system, are two national referral hospitals and two large private hospitals that offer a range of specialist health care. Figure 1, is a schematic presentation of the MOH organizational service delivery structure [15].

Private sector providers are mainly concentrated in urban areas, where they cater for the population with the capacity to pay, while those in the rural areas are chiefly served by public sector providers [18]. Within the public sector, particularly in the peripheral areas, some of the key challenges that are often cited include, lack of adequate infrastructure, limited access to essential medicines and other health technologies, among others. Within the public sector, access to essential medicines is guaranteed by the government whereas in the private sector, clients pay for medicines through medical insurance or out-of-pocket [15, 18]. However, for a number of chronic medications, the government has entered into partnership with the private outlets (starting with major urban centers) to dispense medicines to patients from the public sector.

Financial resources for health are mainly drawn from government, donors and private sector (including household) sources [15, 18]. For government as a source, a portion of tax revenues is allocated for health service provision in accordance with the national health policy guidelines. Government contributes the bulk of resources, with the private sector playing an increasingly important role in contributing approximately 25% of the total health expenditure. Donor funding on the other hand is mainly focused on specific health program areas such as HIV/AIDS and tuberculosis [18, 19].

Both the government and the private sector also play a significant role in pooling financial resources for health. Through its general taxes, the government raises revenues which it earmarks a proportion to finance health service delivery. At the same time, government as an employer has a medical scheme for its employees, which covers approximately 55% of the public sector employees which translates to approximately 70,000 principal beneficiaries. The scheme is voluntary with the government contributing 50% of the premium amount that employees have to pay to become members [18]. In addition to the public sector employee’s health insurance scheme, there are more than 10 private medical insurance companies of various sizes which in total also cover approximately 70,000 beneficiaries. Therefore, in total there are approximately 140,000 principal beneficiaries with health insurance coverage for a population of about 2.2 million people [18, 20].

With this arrangement, Botswana has made great strides in critical areas such as HIV/AIDS and maternal and child health. For example, the country has recorded universal coverage with key interventions such as antiretroviral treatment (ART) and prevention of mother to child transmission (PMTCT) of HIV. Priority services such antenatal healthcare, skilled birth attendance and childhood vaccination have also consistently recorded high coverage across the country over time [15, 21].

However, as Kutzin points out, UHC should be viewed as a direction rather than a destination [7]. This is particularly true for Botswana when considering the other priority areas such as non-communicable diseases that have not been comprehensively addressed, despite concerted efforts [2, 5, 21].

### Analytical framework

**Fig. 2:**
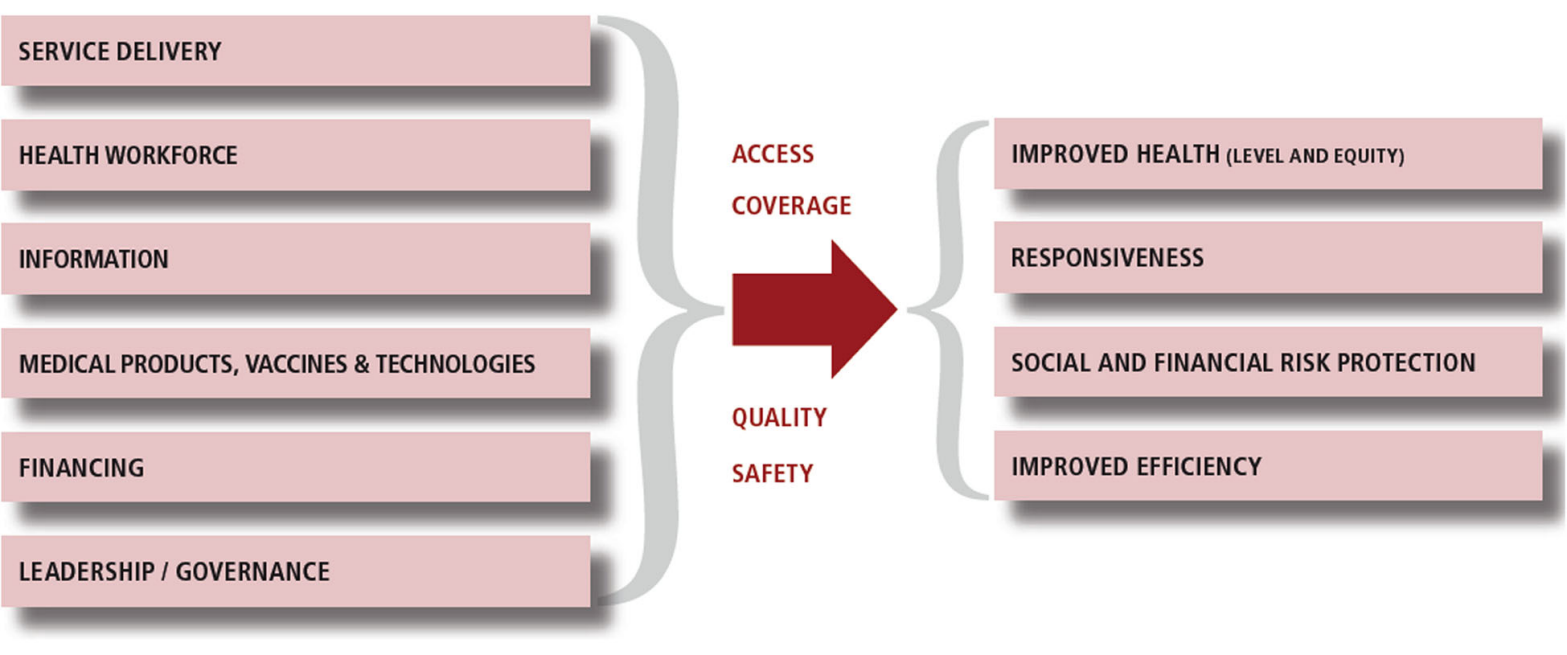
The Health System Framework. An illustration of the health system framework, adapted from the WHO. Everybody’s business: strengthening health systems to improve health outcomes: WHO’s framework for action. Geneva, Switzerland: World Health Organization; 2007

Our analysis was anchored on the framework described by WHO, where the health system is viewed as comprising six discrete pillars [22–25]. This definition was essential in promoting a common understanding among the stakeholders. As shown in Fig. 2, the framework is underpinned by the core functions of the health system, namely; service delivery; health workforce; health information; medical products, vaccines and technologies; financing; and leadership and governance. The framework espouses a logical pathway from inputs into a health system to produce the desired impacts in the form of population health gain [23, 25].

Effective health systems are expected to increase population access to quality medicines and other health interventions in order to improve health [26]. Apart from improving health, the health system is also expected to enhance the responsiveness to the legitimate non-health expectations of those seeking health, and ensure fairness in financial contribution [22, 23, 25]. Responsiveness captures the aspect of the individual’s interactions with the health system, with the considerations of dignity, respect and freedom of choice for those seeking healthcare. Financial and risk protection, on the other hand, covers the fact that contributions to the health system should be based on household ability to pay, and therefore that poor households should not be impoverished in their quest for quality health care. Finally, service provision should be done efficiently and cost effectively while adhering to the principle of equity [23–25].

### Sampling and data collection

The study period was from June to September 2015. Study participants were selected through non-probability sampling procedures termed purposive sampling. In summary, purposive sampling techniques involve selecting certain units or cases based on a specific purpose rather than randomly. In our case, this was a judgement selection based on the participant’s knowledge and involvement in the financing and health service delivery with the Botswana healthcare system. Our sample was then supplemented using snowball sampling methods (also referred to as chain sampling), whereby the initial respondents referred us to the other potential respondents until no new information was forthcoming, or achieved saturation.

In order to guide our initial judgement in the selection of initial respondents, we first undertook a desk review mapping out the different stakeholders involved in health financing and service delivery in Botswana. These comprised of the public and private sectors; non-governmental and civil society organizations (NGO), including the faith based actors; and bilateral and multilateral development organizations, among others. A representative list of 31 organizations was drawn to ensure that views of all key stakeholders were represented in our study.

Of the 31 organizations, 8 were identified as public sector; 11 were from the private sector; 6 were classified as NGOs; and 6 fell into the bilateral and multilateral development organizations. The public sector consisted of employers; health service providers such as hospitals and clinics; and academic and research institutions. Meanwhile, the private sector had an array of providers (mainly private hospitals and private practitioners), health financiers, as well as small-to-large employers.

From the 31 organizations, 1-2 key informants were identified based on their organizational functions and knowledge of health financing and service delivery in the country. In total, we identified a sample of 42 participants, of which 15 were not able to participate due to work commitments. However, from the initial 27 participants, we were able to get 10 additional referrals that enriched our data collection to achieve saturation. Table 1, shows the breakdown of the 37 key informants, ranging from policy-makers to frontline health workers, that were interviewed. The interview process used a semi-structured interview guide developed to solicit perceptions on the impact of health financing reforms on universal health coverage aspirations. Its development was primarily based on prior research on the health system framework and the interaction of its various components as it pertains to progress towards universal health coverage.

**Table 1:** Categories of Participants

Category of participants	Number of Participants
Policy makers	5
Public health providers	7
Private health providers	6
NGO representatives	5
Researchers	4
Health insurance providers	5
Multilateral organizations	3
Bilateral organizations	2
Total	37

The key informant interview (KII) approach was used to collect data from the selected participants. Simply, KIIs are qualitative, in-depth interviews of respondents selected for their first-hand knowledge about a topic of interest. In our case, arrangements were made to secure a 45-min appointment and a suitable venue to conduct the face-to –face interview. This ensured that participants were not unduly distracted during the interview. Before the KII started, researchers introduced themselves, explaining the objectives of the study and securing verbal informed consent to proceed with the interview. Participants were made aware that they could cease participating in the interview at any stage without prejudice.

The interview proceeded with the participant introducing himself or herself and giving an overview of his/her work experience as it relates to the objectives of the study. Leading questions, prepared by the authors, ensured that the participants responded to the key topical issues of interest. The specific focus was on access to medicines, efficiency, and cost-effectiveness of health service delivery. Table 2, shows the summary of some of the open ended questions that participants were asked during the interview. All the interviews were conducted in English.

**Table 2:** Summary of the Interview Questions

1. If the government made it compulsory for public sector employees to join the public employees medical scheme; in your opinion, what would be the potential impacts on the following areas of the health system:
a) Level of financial resources to purchase essential health goods and services for different population groups?
b) Access to and utilization of essential medicines and other health technologies by different population groups^a^?
c) Quality of health goods and services available within both the private and public sectors?
d) Efficiency in resource utilization within the health system to produce the desired health outputs?
e) How cost-effectively the health system selects interventions to meet the population health needs?
2. Overall, what are the potential merits and demerits of this proposed policy reform in the effort towards universal health coverage?
3. What can health system stewards do to mitigate the potential negative impacts of the proposed reform?

^a^Further clarification was provided to include comparisons between the urban –rural populations; those using private-public sector facilities; those employed and unemployed

### Data analysis

Interviews were transcribed verbatim and in their entirety. In order to achieve uniformity, the same researcher (AL) transcribed all the interviews. The transcripts were then analyzed applying thematic content analysis. This approach is appropriate for semi-structured expert interviews as it is used for coding text with a predefined coding system which can then be refined and completed with new themes emerging. Our initial coding system identified, “access to medicines”, category which was defined following priori literature on health systems efforts towards universal healthcare coverage. It is generally accepted that access and utilization of medicines and other health technologies is an essential link between health resources and improvements in population health [4, 6].

From the interview transcripts, data were both deductively and inductively coded, whereby a series of codes were developed and then grouped into similar concepts. These concepts were then combined to form categories (efficiency and cost effectiveness) or were assigned to the category of “access to medicines”, defined earlier. Table 3, shows the successive steps of data reduction employed from simple codes to global themes.

**Table 3:** Thematic Analysis Framework

Codes	Emerging Themes	Global Themes
Government alone cannot meet all the healthcare needs	Partnership between sectors essential to promote access to medicines and other essential health technologies across the population	Promoting access to medicines vital for progress towards universal health coverage
There are inequalities particularly given the rural population is underserved		
Increased insurance coverage in rural areas will attract private sector participation		
Increased insurance coverage leads to better access and use of services	Health insurance coverage vital to mobilize resources to promote access towards universal health coverage	
Private sector providers could play an important role in advancing access		
Public health facilities often experience stock outs of essential medicines		
Capacity to pay will translate to increasing demand for medicines		
Availability and affordability of some of the essential medicines remains a challenge		
There is a gap in access and quality between private and public sectors particularly in terms of access to health technologies	There are inequalities within the health system	
Health insurance could lead to wastage if not properly regulated	If not properly regulated expansion of health insurance coverage could lead to wastage	Efficiency and cost effectiveness in resource utilization vital for universal health coverage
Health insurance market fragmented leading to high costs		
Better pooling and purchasing could improve access and outcomes	Effective pool and strategic purchasing essential for universal health coverage	
Effective and progressive policies and regulation needed to performance of health systems and improve health		
Differential health insurance coverage particularly among employed and unemployed could exacerbate equalities	There is need for effective monitoring and accountability systems in place	
Accountability essential to ensure progress		

## Results

Participants noted that considering the interlinkages across the health system, expansion of the health insurance scheme for public sector employees would reverberate across the entire health system. Many respondents identified that this would be characterized by increased access and utilization of health services among those covered. In addition, participants identified that financial resources to spend on health service delivery as a result of increased employee contributions as well as the government subsidy, would increase substantially offering more options to improve population health in general. However, respondents cautioned that failure to have a holistic view and appreciate this reform within the broader health system context and its interaction with the general population would risk compromising the gains and be potentially counterproductive in the pursuit of UHC in Botswana. We summarize the emergent policy implications focusing on access to medicines, efficiency, and cost-effectiveness of service delivery.

### Access to medicines

Participants postulated that as more financial resources became available, the demand for and utilization of medicines and other health technologies would increase among those with health insurance coverage. This was because, the public sector employees and their beneficiaries who would normally seek healthcare through public health facilities, would have the option to access the private sector health facilities where such health technologies are readily available. In addition, having health insurance often comes with an entitlement where beneficiaries are more prone to demanding specific health goods and services from their providers. Majority of the respondents agreed that, given the profit incentive, providers in the private sector are more likely comply with such specific demands unlike their public sector counterparts, where such incentives do not exist and access to specialized health technologies are largely available through a complex referral system.

Respondents further suggested these market dynamics would attract more private sector participation in the medicine access value chain, even to some small towns and rural areas where civil servants have been deployed, with the view of making a profit. Many of those interviewed felt that this presented opportunities for both increased collaboration and a level of healthy competition among health providers as well as challenges and risks that have to be anticipated and managed.

In terms of potential benefits, some participants suggested that the proposed policy reform could catalyze meaningful collaborations between the public and private sectors that could lead to increased access to medicines and other essential health technologies to a wider population, particularly in the rural areas. It was proposed such partnerships could take various forms such as contracting or outsourcing various services linked to medicine and health technology access, with the aim of increasing effective coverage at the population level. Specifically, some of the respondents felt that the private sector could be incentivized to set up medicine outlets or diagnostic facilities in the new markets where the public sector has limited reach. In addition to serving the civil servants with health insurance coverage in these new markets, these could also be contracted out to serve public sector clients without health insurance at preferential rates. Majority of the participants were in agreement that these measures could greatly contribute to reducing urban-rural inequalities. In fact, it was revealed by a respondent from the public sector that this model was already being implemented on a pilot basis in an attempt to increase access to chronic medications in some locations. Many of those interviewed felt that the significance of such partnerships would increase as health systems reorient themselves to address the emerging epidemic of non-communicable diseases.

*“Already we are piloting various ways to work with private sector, starting with big cities. We plan to increase access to medicines for diabetes and hypertension … and we have started seeing benefits that include decongestion (at the public health facilities) and improved supplies. This approach will become even more important as more people get health insurance throughout the country.” (Public sector participant)*

However, it was noted with caution by some respondents, that unless there are deliberate policy measures and accountabilities to guide the proposed health financing reform to ensure that it is congruent with UHC objectives, the larger part of the population would still face challenges in accessing medicines and other health technologies. In fact, a participant from the private sector hospital cautioned that health inequalities would be exacerbated if there were no measures in place to enable the larger part of the population without health insurance to access essential medicines and other health technologies through private outlets in areas where the public sector has limited reach.

It was further emphasized by some respondents that the risk of having a two tier health system where the public sector is left to serve the unemployed section of the population that does not have health insurance and the private sector predominantly serves the insured group would go against the ideals of UHC. Respondents further indicated that the perceived or real quality implications of such a two tier health system phenomenon would be counterproductive in the overall UHC pursuit. Here quality encompassed key desirable attributes such as safety, efficiency, effectiveness of interventions, responsiveness (person centeredness), and timeliness.

Despite the potential challenges, majority of the participants concurred that there were many benefits that could be harnessed from the proposed policy reform if handled carefully. Obviously, this was predicated upon effective partnership between the public and private sectors operating within a sound regulatory framework. Specifically, a respondent from a multilateral organization noted that there could opportunities to improve logistic management systems by leveraging the private sector competencies, with areas such as procurement, warehousing and distribution of essential medicines in focus. In addition, by shifting a significant proportion of patients (those with insurance cover) to receive services from the private sector, the pressure on the public sector procurement and distribution channels could be relieved, translating to fewer delays and stock-outs at service delivery points. Invariably, this would translate into improvements in the quality and coverage of essential medicines within the population.

*“The public sector alone cannot cope with the demands of health service delivery. They simply do not have the capacity considering the increasing demand.” (Multilateral organization participant)*

In addition, respondents suggested that competition between the public and private sector providers, could act as a catalyst for the health system to focus on efficient and cost-effective delivery. This could lead to reductions in the cost of medicines and overall improvement in the quality of health products available in the market. However, participants further cautioned that this would only be possible with appropriate incentives and accountabilities encouraging health system decision-makers at all levels to reduce wastage, ensure quality and value for money. If such measures were not in place, such competition between the public and private sectors might be counterproductive. Therefore, majority of the respondents observed that it is profoundly important to have policies and regulations that proactively support access to medicines within the pluralistic framework of UHC.

Those interviewed also identified that expansion of health insurance coverage should be coupled with strategic purchasing which prioritizes value-based patient outcomes instead of quantity of interventions and services in order to make a meaningful impact on population health. Participants suggested that measures such as capitation at the primary health care level or some form of case based payment system could reduce the incentive for over-servicing among health providers that are normally reimbursed on a fee-for-service basis. Furthermore, it was emphasized by a respondent from the health insurance industry that large medical pools have the ability to effectively negotiate better prices for medicines and actively encourage the use of cheaper and effective generic medicines where appropriate, and this would have a net-positive impact on improving access to medicines at the population level.

### Efficiency and cost-effectiveness

Majority of participants observed that the proposed policy reform would lead to the creation of a larger health insurance pool that conferred advantages of economies of scale, with better financial risk protection for its membership. However, given its narrow focus on public sector employees, many felt that it would have an insignificant impact on the population level, unless there were sound policies and regulations that seek to promote equity and accountability within the health system. Equally important are the mechanisms to implement and enforce those regulations such that they coherent with the desirable health system objectives of UHC.

In terms of specific benefits, many of those interviewed recognized that expansion of the public sector medical scheme could effectively translate to lower administrative costs, better bargaining and strategic purchasing options from providers, and effective risk sharing, all factors that enhance efficiency and cost-effectiveness within the health system. A participant from the health insurance industry further explained that by pooling risks and resources the large, unpredictable individual financial risks could become more predictable and distributed among all members of the pool.

Therefore, majority of the respondents felt that despite its potential drawbacks, the proposed measure provided the health system with a practical and more efficient option of pooling resources in comparison to the current highly fragmented insurance market. Respondents further clarified that insurance market fragmentation is inefficient and difficult to sustain within any health system and often runs counter to the UHC direction. Inadequate risk pooling, high administrative costs, adverse risk selection and low reserves to effectively deal with financial shocks from large claims are some of the challenges that participants identified as characteristic of highly fragmented health insurance markets. Therefore, participants suggested that through deliberate legislation or market dynamics or a combination of both, efforts towards consolidation in the health insurance market could enhance efficiency. However, there was caution that regulators ought to guard against monopolistic tendencies that could emerge if only one player were allowed to dominate a certain key market.

It was a prevailing view among participants that having health insurance comes with an entitlement whereby individuals and households are likely to be aware and demand for health services. Therefore, many respondents cautioned that it was necessary to carefully balance access to and utilization of health services with appropriate accountability and cost-curbing measures such as copayments to discourage the potential moral hazard that could lead to misuse. In addition, majority of participants suggested that reimbursement options should be carefully planned to reduce the temptation towards over-servicing by health providers and should prioritize value-based patient outcomes instead. This approach could lead to improvements in effective coverage with essential health services without unnecessary and wasteful cost escalations.

During the interviews, a participant from the insurance industry pointed out that a large health insurance pool could also have a set of tools, such as strategic purchasing of health services that could be easily applied to ensure that their beneficiaries receive effective health interventions at the lowest possible cost. For example, using cheaper and effective generic medicines instead of branded ones with similar outcomes. In addition, some participants suggested that, through appropriate incentives such as premium rebates, large insurance schemes could play a crucial role in promoting cost-effective public health interventions such as smoking cessation and adoption of other healthy lifestyles in order to avoid expensive medical treatments. On the contrary, small insurance schemes do not often have adequate fiscal space to offer such incentives to their members that could encourage positive behavior.

*“(Health) insurance companies can play an active role in health promotion by getting their clients to take up healthier lifestyles like going to the gym. Actually some pay for such membership which is good for the health of their clients.” (Private sector participant)*

Furthermore, majority of the participants felt that larger health insurance pools could have the capacity to effectively play a gate-keeping role by ensuring that patients move up the referral chain based on need and not demand; such that only those needing advanced specialist care have access to such services. A participant from the insurance industry explained that this could be achieved by contracting providers and standardizing practice across board, in terms of utilization of expensive diagnostic technologies, prescription practices and procedures with the aim of ensuring quality, efficient and cost-effective delivery. In addition, the larger the pool, the more leverage there is to negotiate better prices for different health services, medicines and other health products. Invariably, this would translate to more efficient and cost-effective health service delivery in contrast to smaller pools or individual purchasers that would not necessarily have such leverage.

However, a number participants still expressed concern that the proposed health financing reform will have a negligible impact in terms of the country’s UHC aspirations, largely because of its narrow focus on the public sector employees. Majority indicated that overall, UHC would only become a reality when the larger part of the population that is unemployed could also have equal access with their employed counterparts. Table 4, summarizes some of the key merits and demerits identified by the participants.

**Table 4:** Advantages and disadvantages of the proposed health financing reform

Advantages	Disadvantages
Potential to increase financial resources available for purchasing of health goods and services	Narrow focus on public sector employees that is likely to worsen the existing inequalities when considering the whole population
Larger health insurance pool with better risk sharing and cross subsidization among those covered	Real risk of establishing a two tier health system that would be counterproductive to the UHC ideals (particularly in terms of quality of services)
Larger pool with lower administration fees; better opportunities for strategic purchasing to enhance efficiency, cost-effectiveness and ensuring value based outcomes	Without adequate regulation and accountability, the proposed policy reform could potentially lead to cost escalation in health service delivery due to factors such as over servicing by health providers and monopolistic tendencies
Provides opportunities to address urban-rural inequalities through innovative public private partnership in service delivery as well as improvements in health system responsiveness	
Provides a platform for health system stewards to expand health insurance across the population through deliberate policy decisions	

## Discussion

Any health system aspiring towards UHC must strive to have adequate financial resources to pay for health service delivery [4, 6, 24]. For many decision-makers, expansion of health insurance coverage, particularly among those in formal employment, has become an attractive first step [4, 8, 27, 28]. However, taking a broader health system view, it is increasingly clear that the process of expanding health insurance coverage can be fraught with many challenges and unintended consequences that can derail progress towards UHC [8, 27–30].

**Fig. 3:**
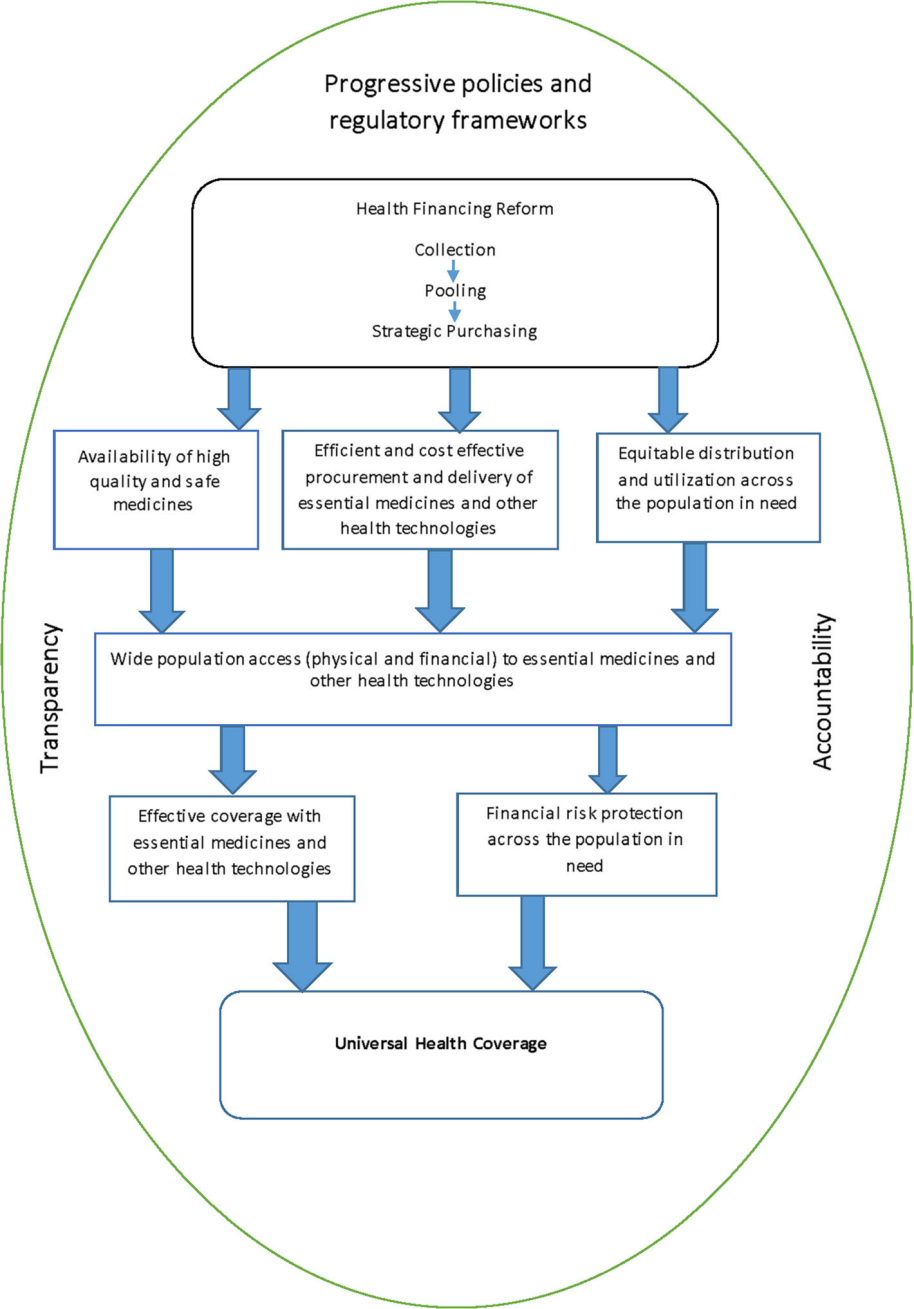
Pathway to Universal Coverage. An illustration showing the pathway from health financing reform to universal coverage with essential medicines and other health technologies

Kutzin [7] cautions that health financing reforms alone do not necessarily translate to UHC, in as much as they are a necessary ingredient to achieve the goal. He further opines that countries cannot simply spend their way to UHC, but equity, efficiency, cost-effectiveness, transparency and accountability within the health system must be prioritized in order to make progress towards UHC. Figure 3, is a schematic illustration [4, 7, 25], showing the pathway (with intermediate steps) from health financing reform to universal health coverage.

In fact, as previously mentioned some reforms implemented in the name of expanding coverage have led to exacerbation and entrenchment of inequalities in some settings [7–11]. However, Tangcharoensathien et al. [12] and Lagomarsino et al. [13] in their analysis of various attempts to expand health insurance coverage in low- and middle- income countries, established that despite the many challenges, progress has been realized in many settings. Their analysis further underscores the fact that there is no single prescriptive pathway towards UHC, but each country must adapt to its own circumstances.

Our study contributes substantially to this debate by taking a Botswana health system view to distill relevant information that could be useful to decision-makers implementing health financing reform in low- and middle- income countries. In our analysis, we have paid specific attention to the key health system topics of access to medicines, efficiency and cost-effectiveness, given their significance and relevance in solving the hurdles that many health systems in low- and middle- income countries face along their path to UHC [4, 31].

Cognizant of the potential limitation of basing our findings on observations of a small sample of participants, we made efforts to have a diverse group drawn from different stakeholders of the health system such that the discussion was rich and informative. In addition, qualitative studies offer the benefit of a deeper investigation into important policy matters and perceptions that could be concealed through simple aggregation methods.

Invariably, expanding the public sector employees’ health insurance offers an easy and practical way to increase the financial resources available to pay for essential medicines and other health services [4, 29]. However, health financing does not act alone to achieve all the attributes necessary for UHC, such as improvements in effective coverage and financial risk protection for the whole population. Deliberate policy and implementation actions that mainstream equitable distribution and utilization of medicines, technology and other essential services across the health system are vital to ensure that health financing reforms translate to overall population health gains [7, 31, 32]. Therefore, expanding health insurance coverage within a small section of the population (such as public sector employees), could potentially increase or entrench existing inequalities.

On the other hand, health insurance coverage comes with an entitlement where beneficiaries are proactive in demanding health goods and services [8, 23, 27–29]. Therefore, as the proportion of those insured increases, other key components of the health system particularly medicines and health technologies should be prepared to cope with the increased demand. Removing the financial barriers that largely constraint utilization among those in peripheral areas, might not necessarily lead to increased utilization since the services in demand might not be physically in place. However, this provides a good opportunity for private sector and other actors including NGOs to contribute substantively in enhancing service provision in the country, by investing in those new markets where services are in demand [8, 26].

In fact, with proper regulation, public private partnerships in service provision, could offer a practical way through which decision makers could improve the performance of their respective health systems in improving access and bridging the existing inequalities between urban and rural areas. Invariably, when financial access barriers have been minimized through the expansion of health insurance coverage, physical access to medicines, technology and other essential services is a challenge that could be effectively addressed by such collaborative partnerships. Furthermore, considering that with health insurance, money practically follows the patient, responsiveness of the health system is likely to improve with the increased competition among providers.

The narrow focus on public sector employees alone in the proposed policy reform, poses significant challenges that health systems stewards need to recognize and mitigate accordingly. There is a real risk of exacerbating the existing inequalities between those employed and the unemployed section of the population, unless there are deliberate steps to innovatively extend coverage to the rest of the population. For instance, the employed group would be able to access and utilize services from both the public and private sectors while those who are unemployed would be constrained to the public sector. The risk of creating a two tier health system, where there is an oversupply of expensive and hi-tech services in the private health sector serving the rich and powerful class and the public health sector serving the unemployed would be counterproductive in progress towards UHC [4, 7, 12, 13].

Furthermore, given that the government subsidy for the public sector employees who join the insurance scheme comes from general tax revenues; many would argue that this would be tantamount to taxing the poor (who are the majority) and subsidizing the rich. Therefore, unless there are deliberate efforts to extend coverage to include those who are unemployed, marginalized and indigents, the proposed policy reform might be regressive to the overall UHC aspirations.

Despite, these potential challenges, a larger health insurance pool would have many advantages, such as the capacity to spread risks across a large membership base, to incur lower administrative costs, and bargain for lower tariffs from health providers. In addition, large pools have the leverage to incentivize health providers to focus on value based outcomes instead of quantity of services as well as promote the use of cheaper and effective medicines and technologies rather than expensive ones [4, 29, 31]. All these, if properly harnessed, could be vital to enhancing efficiency and cost-effectiveness in health service delivery.

Therefore, the role for effective regulation and enforcement cannot be overstated in any health financing reform. There should be measures in place aimed at strengthening governance and accountability structures within both public and private sectors to ensure that all stakeholders adhere to the ideals of quality, efficiency and cost effectiveness. A focus on equity in health and finance is essential to ensure that there is population risk sharing and cross subsidization. Failure to recognize and address such market dynamics could derail progress towards UHC [4, 28–30].

## Conclusion

Overall, the features that characterize the pathway towards UHC are not necessarily linear, but require an adaptive outlook that balances various health systems’ objectives and demands in order to maximize population health at the lowest possible cost [4, 6, 25]. Therefore, it is vital that the policy objectives of any proposed health financing reform be clearly defined and with a pragmatic intent on how various developments fit to the overall UHC goal. It is only through this approach that decision-makers would be able to optimize the gains and mitigate risks accordingly. Failure to do this could result in negative unintended consequences that would put the overall reform in jeopardy [22, 27–31]. Health system stewards must judiciously apply the tools of regulation and accountability to ensure that they steer the health system toward achieving its intended objectives of maximizing population health in an efficient and cost-effective manner [23, 24].

## Abbreviations

MOH: Ministry of health
NGO: Non-governmental organization
PHC: Primary healthcare
PPP: Public private partnership
UHC: Universal health coverage
WHO: World health organization
